# COVID-19 vaccination rates as a proxy for health system resilience: implications of development disparities for cervical cancer control during the pandemic

**DOI:** 10.3389/fmed.2026.1858206

**Published:** 2026-07-24

**Authors:** Qian Guo, Chen Xu, Jia Li, Chunlei Qi

**Affiliations:** 1 Department of Obstetrics and Gynecology, the First Affiliated Hospital of Air Force Medical University, Xi’an, Shaanxi, China; 2 Department of Anesthesiology, Xi’an Electric Power Central Hospital, Xi’an, Shaanxi, China

**Keywords:** cervical cancer, COVID-19, COVID-19 vaccination, global burden of disease, health system resilience, human development index

## Abstract

**Purpose:**

To assess COVID-19’s impact on global cervical cancer burden and examine how COVID-19 vaccination rates and Human Development Index (HDI) interact to shape disease trends, with exploratory projections to 2050.

**Methods:**

Using Global Burden of Disease 2023 data (1990–2023), we analyzed age-standardized incidence (ASIR) and disability-adjusted life-year (DALY) rate of cervical cancer. Joinpoint regression estimated annual percentage changes (EAPC) for pre-pandemic (1990–2019) and pandemic (2020–2023) periods. Linear and interaction models assessed associations between HDI, COVID-19 vaccination rates, and EAPC, with bidirectional stratified analyses. Projections to 2050 were generated under three exploratory scenarios.

**Results:**

The pandemic coincided with a reversal of the pre-pandemic declining trend in cervical cancer burden indicators. Global ASIR increased 6.0% (*p* = 0.031), with EAPC shifting from −0.41%/year (*p* < 0.001) to 3.93%/year (*p* = 0.008). Low-SDI regions experienced the sharpest rise (ASIR 18.6%). EAPC showed a significant negative association with HDI (*β* = −15.4, *p* < 0.001). The interaction term was positive (*β* = 36.1, *p* < 0.001, Δ*R*^2^ = 6.4%). Bidirectional stratification revealed divergent patterns. In low-HDI countries, higher vaccination coincided with lower EAPC (*β* = −7.15, *p* = 0.005). In high-HDI countries, a positive association emerged (*β* = 6.53, *p* < 0.001). HDI effects attenuated across vaccination quartiles: strong negative associations in low-vaccination contexts (Q1: *β* = −29.06, R^2^ = 39.7%) to non-significance in high-vaccination settings (Q4: *β* = −4.97, *p* = 0.244). Under current trends, low-SDI regions would achieve WHO 90–70-90 targets ~10–12 years behind high-SDI regions.

**Conclusion:**

COVID-19 vaccination rate shows context-dependent associations with cervical cancer burden: stronger negative associations in low-HDI settings and positive associations in high-HDI settings. These findings support dual-purpose investments in emergency infrastructure for pandemic preparedness and cancer control.

## 1 Introduction

Cervical cancer ranks fourth among cancers in women worldwide (1). In 2022, there were approximately 660,000 new cases and 350,000 deaths, with 90% occurring in low-and middle-income countries (2). The disease is largely preventable through HPV vaccination, screening, and early treatment (3). In 2020, the World Health Organization (WHO) launched the “Global Strategy to Accelerate the Elimination of Cervical Cancer” (4). The strategy sets 90–70-90 targets to be achieved by 2030: 90% HPV vaccination, 70% screening, and 90% treatment access. Importantly, these are rate targets. Elimination—defined as an incidence below 4 per 100,000 women—will take decades longer (5). Before COVID-19, global cervical cancer age-standardized incidence rate (ASIR) and disability-adjusted life years (DALYs) had been declining steadily, driven by screening and HPV vaccination (6).

The COVID-19 pandemic disrupted health systems worldwide. Resources shifted away from routine services, including cancer prevention (7). Screening volumes dropped by 40–60% in many countries during the first pandemic year (8). Studies from England, Denmark, and the United States confirmed significant service interruptions (9–11). However, these studies were limited to high-income countries and focused on short-term disruptions. Two critical gaps remain. First, few studies have quantified whether these interruptions actually increased cervical cancer incidence and DALYs at the global level. Second, existing explanations for recovery disparities—such as “political will” or “health investment”—resist cross-country comparison, leaving unidentified the systemic factors that enabled some countries to maintain services while others did not.

Health system resilience—the ability to maintain essential functions during crises—may explain these differences (12). Resilient systems have strong infrastructure, adequate workforce, and effective governance (13). However, resilience is hard to measure across countries, especially in low-resource settings with incomplete data (14). The COVID-19 vaccination campaign offers a measurable window into health system crisis response. The same infrastructure and workforce deployed for vaccination—cold chains, community health workers, supply chains—are required to sustain cervical cancer screening. Thus, vaccination coverage serves as a proxy indicator of emergency mobilization capacity (15, 16). Thus, we use COVID-19 vaccination rate as a proxy indicator of health system resilience (17). We also include the Human Development Index (HDI)—a composite of life expectancy, education, and income—to account for structural development (18). The Socio-demographic Index (SDI) provides a regional stratification framework used in Global Burden of Disease (GBD) analyses (19, 20).

To test whether this proxy captures meaningful variation in pandemic resilience, this study evaluates global cervical cancer burden from 1990 to 2023. We quantify changes in ASIR and DALYs between pre-pandemic (1990–2019) and pandemic (2020–2023) periods. We examine how HDI and vaccination rate relate to burden trajectories—separately and jointly—and whether higher vaccination attenuates the HDI–burden association in resource-limited settings. We also project exploratory scenarios to 2050.

## 2 Methods

### 2.1 Study data and population

This study utilized the GBD 2023 database, accessed via the Global Health Data Exchange (GHDx) platform.^1^ The GBD database provides comparable estimates of mortality, incidence, and risk factors across 204 countries and territories, stratified by regional and SDI categories.

We extracted annual estimates for three core indicators of cervical cancer burden from 1990 to 2023: age-standardized incidence rate (ASIR), age-standardized prevalence rate (ASPR), and disability-adjusted life-year (DALY) rate per 100,000 population. All estimates were accompanied by 95% uncertainty intervals (UIs), derived from the 2.5th and 97.5th percentiles of 1,000 posterior sampling distributions to account for both sampling and non-sampling errors.

For national-level analyses, 2019 HDI data were obtained from the United Nations Development Program.^2^ COVID-19 vaccination rate data-defined as the percentage of population fully vaccinated against COVID-19 by December 31, 2023-were extracted from the WHO COVID-19 dashboard.^3^

### 2.2 Statistical analysis

To evaluate COVID-19-associated changes in cervical cancer burden, pre-pandemic (1990–2019) and pandemic (2020–2023) values were compared. For global and each SDI stratum, mean ASIR, and DALY rates with 95% CI were calculated. Percentage changes were computed as [(pandemic mean - pre-pandemic mean)/pre-pandemic mean] × 100%. Two-sample t-tests accounting for GBD estimation uncertainty were applied to test temporal differences.

Spatial distributions were visualized using maps. Countries were categorized into five groups based on quintiles of ASIR and DALY rates in each period, ensuring consistent color scales for 2019–2023 comparisons.

Joinpoint regression was used to estimate the annual percentage change (EAPC) in ASIR and DALY rates for the pre-pandemic (1990–2019) and pandemic (2020–2023) periods. We acknowledge that EAPC estimates for the pandemic period are based on only four annual observations (2020–2023). While Joinpoint regression can estimate trends from short series, the resulting *R*^2^ values should be interpreted with caution, as high values may reflect mathematical artifacts of fitting a line to a limited number of points rather than strong model fit. These estimates are presented as descriptive summaries of recent changes rather than stable long-term trends.

Linear regression models were used to examine national-level determinants of cervical cancer burden changes. The dependent variable was the EAPC of DALY rates during 2020–2023. Independent variables included 2019 HDI (continuous, range 0–1) and COVID-19 vaccination rate (continuous, range 0–1), with the interaction term (HDI × vaccination rate) added to test moderation. The unit of analysis was the country (n = 173 countries with complete data). Regression coefficients (*β*) represent the change in EAPC (percentage points per year) associated with a 1-unit increase in the predictor. Model fit was compared using adjusted R^2^ and Akaike information criterion (AIC).

Regression diagnostics were performed for all models. Predictors were mean-centered before constructing interaction terms to reduce multicollinearity; variance inflation factors (VIF) were all 1.0, indicating no substantial collinearity. Residual distributions were examined for normality (Shapiro–Wilk test, *p* < 0.001) and homoscedasticity (Breusch-Pagan test, *p* = 0.157). As residual normality showed minor deviation—not uncommon with this sample size—we used heteroscedasticity-consistent robust standard errors (HC3) for all inference. Influential points were assessed using Cook’s distance (maximum = 0.178; no values > 0.5). These diagnostics collectively confirm the robustness of the regression findings. Detailed regression diagnostic results, including VIF values, Shapiro–Wilk test, Breusch-Pagan test, and Cook’s distance, are presented in Supplementary Table S1. Residual diagnostic plots are shown in Supplementary Figure S2.

Hierarchical regression analysis was employed to examine the interaction effect between the HDI and COVID-19 vaccination rates on disease burden. Firstly, the data were stratified according to the tertiles of HDI (low, middle, and high) and the quartiles of COVID-19 vaccination rates (Q1-Q4), respectively. Within each stratum, simple linear regression models were fitted to estimate the effect of HDI on the EAPC (stratified by COVID-19 vaccination rates) or the effect of COVID-19 vaccination rates on the EAPC (stratified by HDI). Subsequently, a multiple regression model incorporating an interaction term was constructed to validate the moderating effect. The regression coefficients, R^2^, and significance levels for each stratum were reported.

Exploratory scenario modeling was used to project cervical cancer burden to 2050. Projections were generated using age-period-cohort models with three explicitly defined scenarios:

1. Shock lingers scenario: Assumes the pandemic-period EAPC for DALYs (0.91% per year) continues through 2030, followed by a linear recovery to the pre-pandemic EAPC (−0.41% per year) by 2040, which is then maintained. This represents a pessimistic trajectory where health systems in affected regions fail to recover quickly.
2. Status quo remains scenario: Assumes continuation of 2010–2019 trends. This serves as a counterfactual baseline representing the trajectory if the pandemic had not occurred or if systems had recovered immediately.
3. Enhanced intervention scenario: Assumes rapid scale-up of cervical cancer control interventions: HPV vaccination rate increases linearly from 15% (2023) to 90% (2030), screening rate from 40 to 70%, and treatment access from 50 to 90%. Effect sizes were derived from Canfell et al. (21), where each 10% increase in HPV vaccination is associated with a 2.3% reduction in ASIR EAPC and a 3.1% reduction in DALY EAPC. Combined effects were modeled multiplicatively.

All projections were generated globally and by SDI region, with 95% prediction intervals estimated via bootstrapping (1,000 iterations). These projections are exploratory scenario illustrations rather than robust epidemiological forecasts; they are intended to inform policy deliberation by visualizing the range of possible futures under different assumptions.

All analyses were conducted using R software (4.5.2). Statistical significance was set at bilateral *p* < 0.05.

## 3 Results

### 3.1 The unequal impact of the COVID-19 pandemic on the burden of cervical cancer

**Figure 1:**
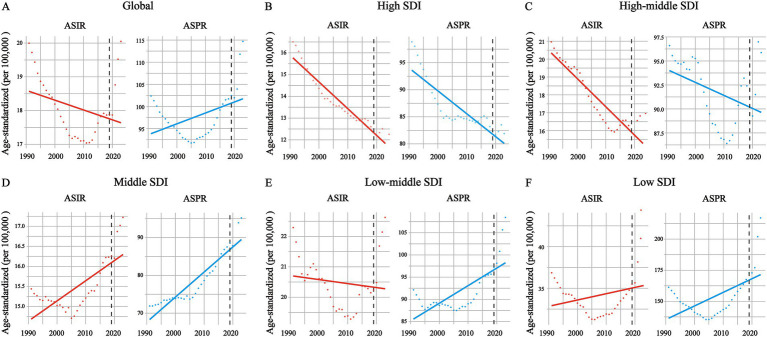
Trend chart of cervical cancer ASIR and ASPR globally and across SDI regions, 1990–2023. **(A)** Global. **(B)** High SDI. **(C)** High-middle SDI. **(D)** Middle SDI. **(E)** Low-middle SDI. **(F)** Low SDI. ASIR: Age-standardized incidence rate; ASPR: Age-standardized prevalence rate; SDI: Socio-demographic Index.

The COVID-19 pandemic has coincided with reversal the declining trend in the global burden of cervical cancer. Figure 1 illustrates the changes in the ASIR and ASPR from 1990 to 2023: high and high middle SDI regions maintained a declining trend, while middle, low middle, and low SDI regions experienced an increase after 2020, with the low SDI region bearing the highest burden and showing the steepest rise. Table 1 quantifies the changes before and after the pandemic (1990–2019 vs. 2020–2023). Globally, the ASIR increased by 6.0% (*p* = 0.031), while the DALY rate remained stable (−1.2%, *p* = 0.677). Stratified analysis revealed significant inequalities: in low SDI regions, the ASIR rose by 18.6% (*p* < 0.001), and the DALY rate increased by 7.0% (*p* = 0.092); in high SDI regions, the ASIR decreased by 12.0% (*p* < 0.001), and the DALY rate dropped by 23.4% (*p* < 0.001). The ASIR also significantly increased in middle SDI (10.0%) and low middle SDI (7.0%) regions. In high-SDI regions, modest ASIR changes concurrent with DALY declines suggest catch-up screening detection rather than true incidence rise; in low-SDI regions, ASIR and DALY increases together suggest true burden increase from sustained disruptions.

**Table 1:** Comparison of cervical cancer ASIR and DALYs before and after the COVID-19 pandemic across global and SDI regions (1990–2019 vs. 2020–2023).

Regions	Measure	1990–2019	2020–2023	Change (%)	*p*
Global	ASIR	17.97 (17.67–18.28)	19.05 (18.12–19.98)	6.0%	0.031
	DALYs	287.68 (279.22–296.14)	284.31 (270.89–297.74)	−1.2%	0.677
High SDI	ASIR	14.01 (13.57–14.44)	12.33 (12.21–12.45)	−12.0%	<0.001
	DALYs	163.38 (154.98–171.78)	125.10 (123.72–126.47)	−23.4%	<0.001
High-middle SDI	ASIR	18.00 (17.38–18.61)	16.51 (15.95–17.06)	−8.3%	<0.001
	DALYs	285.53 (268.61–302.45)	229.09 (223.31–234.86)	−19.8%	<0.001
Middle SDI	ASIR	15.30 (15.15–15.46)	16.83 (16.40–17.26)	10.0%	<0.001
	DALYs	258.54 (254.65–262.44)	254.10 (249.89–258.31)	−1.7%	0.129
Low-middle SDI	ASIR	20.32 (20.04–20.59)	21.73 (20.83–22.63)	7.0%	0.003
	DALYs	391.76 (380.40–403.13)	374.84 (362.80–386.88)	−4.3%	0.045
Low SDI	ASIR	33.37 (32.79–33.95)	39.56 (36.24–42.88)	18.6%	<0.001
	DALYs	685.68 (667.74–703.62)	733.58 (680.86–786.31)	7.0%	0.092

SDI: Socio-demographic Index; ASIR: age-standardized incidence rates; DALYs: disability-adjusted life years. Changes in ASIR during 2020–2023 likely reflect diagnostic delays and screening backlogs rather than true biological incidence surges, given cervical cancer’s natural history.

**Figure 2:**
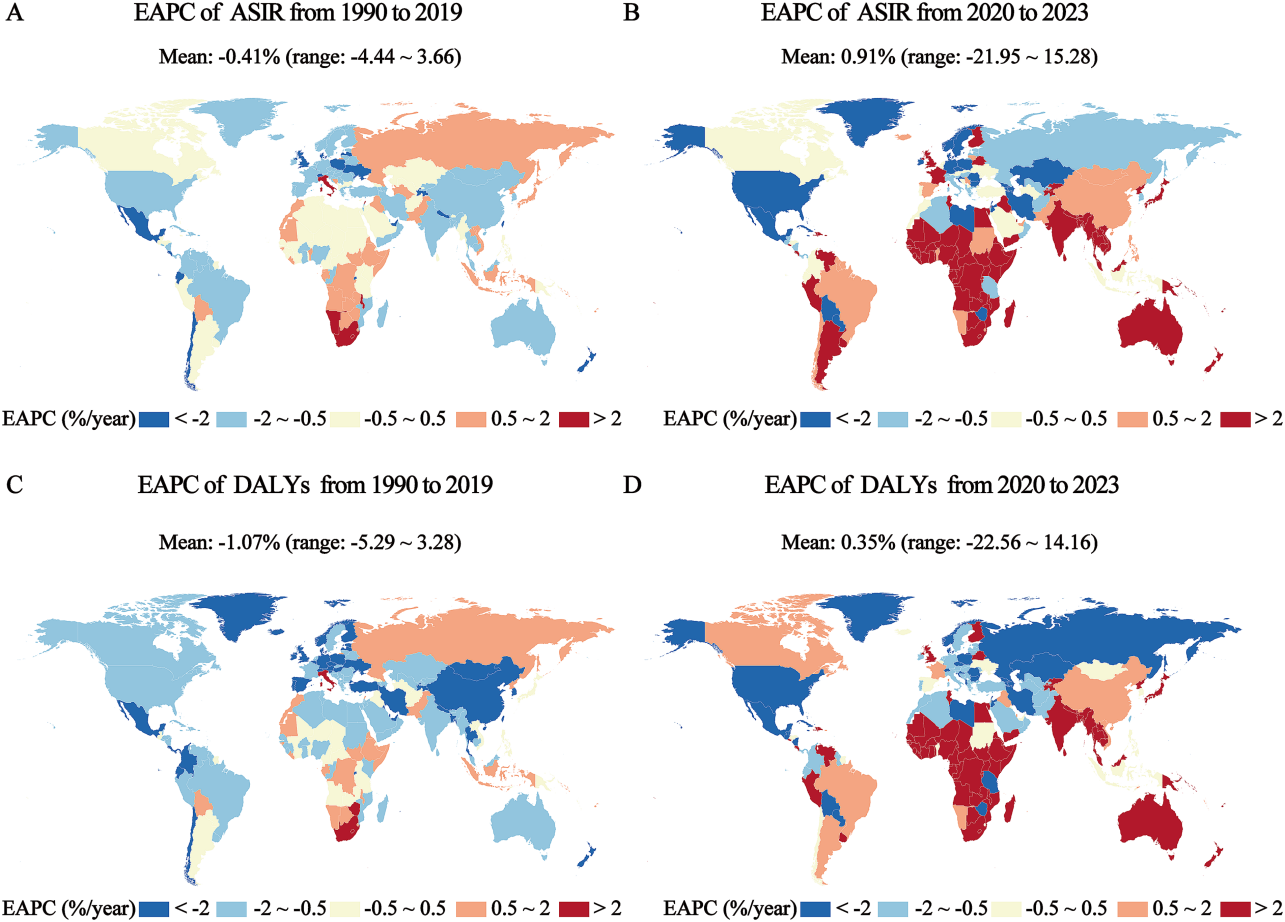
Estimated annual percentage change (EAPC) in cervical cancer burden across countries. **(A)** EAPC in ASIR, 1990–2019. **(B)** EAPC in ASIR, 2020–2023. **(C)** EAPC in DALYs, 1990–2019. **(D)** EAPC in DALYs, 2020–2023. EAPC estimates for 2020–2023 are based on four data points and should be interpreted as descriptive indicators of disruption magnitude rather than robust long-term trends. ASIR: Age-standardized incidence rate; DALYs: Disability-adjusted life years.

Table 2 presents the changes in the EAPC. The global EAPC for ASIR reversed from −0.41% per year (*p* < 0.001, *R*^2^ = 0.57) to 3.93% per year (*p* = 0.008, *R*^2^ = 0.98). The reversal was most pronounced in low SDI regions, where it increased from −0.21% per year (*p* = 0.042) to 6.87% per year (*p* = 0.001). In high SDI regions, the EAPC during the pandemic was 0.20% (*p* = 0.747), showing no significant change. Figures 2, a map, shows that from 1990 to 2019, most countries had negative EAPCs for ASIR and DALYs, but from 2020 to 2023, sub-Saharan Africa and South Asia shifted to positive values, while high-income regions maintained negative values.

**Table 2:** EAPC in ASIR of cervical cancer across global and SDI regions: 1990–2019 vs. 2020–2023.

Regions	Period	EAPC (95% CI)	*p*	*R* ^2^
Global	1990–2019	−0.41 (−0.54 to −0.28)	<0.001	0.57
	2020–2023	3.93 (3.21 to 4.65)	0.008	0.98
High SDI	1990–2019	−0.93 (−1.02 to −0.84)	<0.001	0.94
	2020–2023	0.20 (−0.85 to 1.26)	0.747	0.06
High-middle SDI	1990–2019	−1.05 (−1.16 to −0.94)	<0.001	0.93
	2020–2023	2.51 (1.02 to 4.03)	0.080	0.85
Middle SDI	1990–2019	0.19 (0.09 to 0.29)	0.001	0.34
	2020–2023	1.93 (0.92 to 2.96)	0.064	0.88
Low-middle SDI	1990–2019	−0.30 (−0.42 to −0.18)	<0.001	0.48
	2020–2023	3.25 (2.00 to 4.51)	0.036	0.93
Low SDI	1990–2019	−0.21 (−0.40 to −0.02)	0.042	0.14
	2020–2023	6.87 (6.47 to 7.28)	0.001	1.00

SDI: Socio-demographic Index; EAPC: estimated annual percentage change. EAPC estimates for 2020–2023 are derived from four annual data points. High *R*^2^ values in this short interval may partially reflect mathematical properties of limited observations rather than robust epidemiological trends. These estimates should be interpreted as descriptive indicators of disruption magnitude.

### 3.2 HDI and cervical cancer prevention during the COVID-19 pandemic

**Figure 3:**
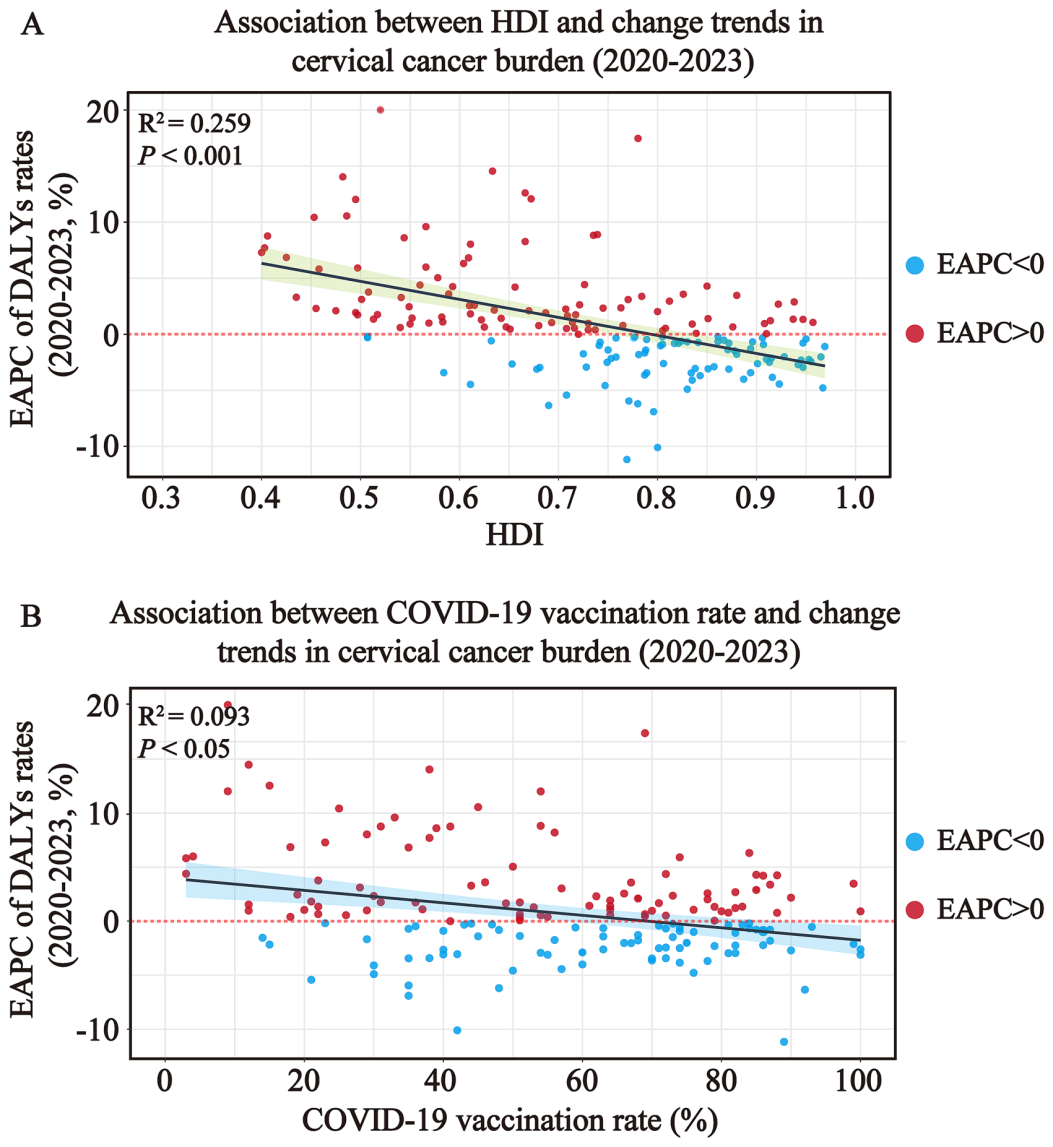
Associations between HDI, COVID-19 vaccination rate, and cervical cancer burden trajectories during the pandemic (2020–2023). **(A)** Association between HDI and EAPC of DALY rates. **(B)** Association between COVID-19 vaccination rate and EAPC of DALY rates. Red points: EAPC > 0 (increased reported burden); blue points: EAPC < 0 (decreased reported burden). HDI: Human Development Index; EAPC: Estimated annual percentage change; DALYs: Disability-adjusted life years.

Figure 3 illustrates the relationships between the HDI, COVID-19 vaccination rates, and the EAPC in DALY rates from 2020 to 2023, respectively. Figure 3A shows that countries with lower HDI predominantly exhibit positive EAPC values (indicating an increased burden), whereas countries with higher HDI mostly show negative EAPC values (indicating a reduced burden). Linear regression confirms a significant negative correlation (*β*: -15.4, 95% CI: −19.3 to −11.6, *p* < 0.001, *R*^2^ = 0.259). Figure 3B demonstrate a significant negative correlation between COVID-19 vaccination rates and the EAPC of DALY rates (*β* = −5.77, *p* < 0.05). However, COVID-19 vaccination rates alone explain only 9.3% of the variation in EAPC (*R*^2^ = 0.093), suggesting that while COVID-19 vaccination serves as an important predictor of pandemic resilience, it is not the sole determinant of disease burden. Notably, the explanatory power of COVID-19 vaccination rates (*R*^2^ = 9.3%) is lower than that of HDI (*R*^2^ = 25.9%).

### 3.3 The interactive effect of HDI and COVID-19 vaccination rates

To test whether vaccination rate modifies the HDI–burden association, we constructed hierarchical regression models. HDI and vaccination coverage were positively correlated (*r* = 0.72, *p* < 0.001), with EAPC direction varying across the distribution (Supplementary Figure S1). Table 3 presents linear regression models testing the interaction between the two factors. In Model 1 (main effects), only HDI is significant (*β*: -15.49, *p* < 0.001), while COVID-19 vaccination rate was not (β: -0.61, *p* = 0.68), with an R^2^ of 0.275. Model 2, incorporating an interaction term, shows a significant improvement in fit (Δ*R*^2^ = 0.064, F change = 16.27, *p* < 0.001), with a total R^2^ of 0.339. The interaction term was positive (*β*: 36.1, 95% CI: 18.43 to 53.75, *p* < 0.001), and both main effects remained significant (HDI: *β* = −35.66; COVID-19 vaccination rate: *β* = −26.19).

**Table 3:** Analysis of the interaction between HDI and COVID-19 vaccination rates, and cervical cancer burden indicators.

Variable	Model 1 (main effect)	Model 2 (interaction effect)
Sample size (N)	173	173
Constant term	20.89 (3.09) ***	26.19 (3.63) ***
HDI (0–1)	−15.49 (−20.17, −10.81) ***	−35.66 (−46.47, −24.85) ***
COVID-19 vaccination rate (0–1)	−0.61 (−3.49, 2.27)	−26.06 (−38.82, −13.30) ***
HDI × COVID-19 vaccination rate	-	36.09 (18.43, 53.75) ***
R^2^	0.275	0.339
ΔR^2^	-	0.064 ***
*F*-value	32.28 ***	28.90 ***
F change	–	16.27 ***

Values in parentheses represent 95% confidence intervals; HDI and COVID-19 vaccination rate are continuous variables (0–1); ***p < 0.001; Δ*R*^2^ significance based on F change test. This model examines ecological associations and cannot establish causality.

**Figure 4:**
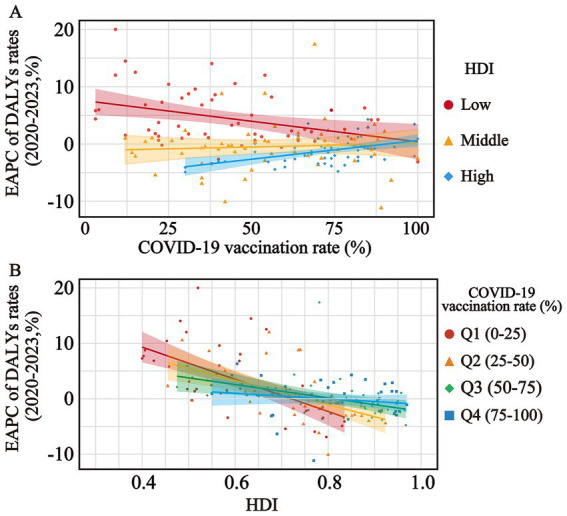
HDI and vaccination rate associations with cervical cancer burden, stratified by development level and vaccination status. **(A)** Association of vaccination rate on EAPC, stratified by HDI tertiles. **(B)** Association of HDI on EAPC, stratified by vaccination rate quartiles. EAPC: Estimated annual percentage change.

Bidirectional stratified analysis (Tables 4, 5) revealed asymmetric associations. Figure 4A shows that when examining the effect of COVID-19 vaccines stratified by HDI, the association between vaccination rate and EAPC diminishes and even reverses as the level of development increases. In low-HDI countries, a 10% increase in COVID-19 vaccination rates significantly reduces the value by 0.715 percentage points (*β* = −7.15, *p* = 0.005), whereas a significant positive correlation is observed in high-HDI countries (*β* = 6.53, *p* < 0.001). Figure 4B shows that when stratified by vaccination rates, the HDI-EAPC association declined stepwise: from *β* = −29.06 in the lowest vaccination quartile (R^2^ = 39.7%) to non-significance in the highest quartile (*β* = −4.97, R^2^ = 3.3%, *p* = 0.244). For each quartile increase in vaccination rates, the HDI effect size declined by 42–58%.

**Table 4:** Association between COVID-19 vaccination rate and EAPC at different HDI levels.

Variable	Vaccination rate effect (β)	SE	95% CI	*p*	*R* ^2^
Low HDI	−7.152	2.466	−12.10 to −2.21	0.005	0.133
Middle HDI	1.288	2.421	−3.56 to 6.14	0.597	0.005
High HDI	6.533	1.737	3.05 to 10.01	<0.001	0.205

HDI: Human Development Index; low HDI: 0.28–0.68; middle HDI: 0.68–0.82; high HDI: 0.82–0.97; SE, Standard Error. Coefficients represent change in EAPC per 1-unit change in vaccination rate. This analysis is ecological; associations at the national level may not reflect individual-level relationships.

**Table 5:** Association between HDI and EAPC at different COVID-19 vaccination rate levels.

Variable (vaccination rate)	HDI effect (β)	SE	95% CI	*p*	*R* ^2^
Q1 (0–25%)	−29.060	5.462	−40.08 to −18.04	<0.001	0.397
Q2 (25–50%)	−16.915	4.665	−26.34 to −7.49	<0.001	0.247
Q3 (50–75%)	−12.074	3.920	−19.99 to −4.16	0.004	0.188
Q4 (75–100%)	−4.970	4.209	−13.47 to 3.53	0.245	0.033

SE, Standard Error. Coefficients represent change in EAPC per 1-unit change in HDI. Q1–Q4 represent quartiles of COVID-19 vaccination rate (0–1 scale). Stepwise attenuation of HDI coefficients across rate quartiles suggests modification of the HDI–burden association by vaccination status.

In low HDI countries, higher COVID-19 vaccination rates were associated with reduced disease burden (*β* = −7.152, 95% CI: −12.10 to −2.21, *p* = 0.005). In high-HDI settings, a positive association emerged (*β* = 6.533, 95% CI: 3.05 to 10.01, *p* < 0.001). HDI effects were strongest in low-vaccination contexts (*β* = −29.06, 95% CI: −40.08 to −18.04, *R*^2^ = 39.7%) and attenuated to non-significance in high-vaccination settings (*β* = −4.97, 95% CI: −13.47 to 3.53, *p* = 0.245).

### 3.4 Projections of cervical cancer burden to 2050

**Figure 5:**
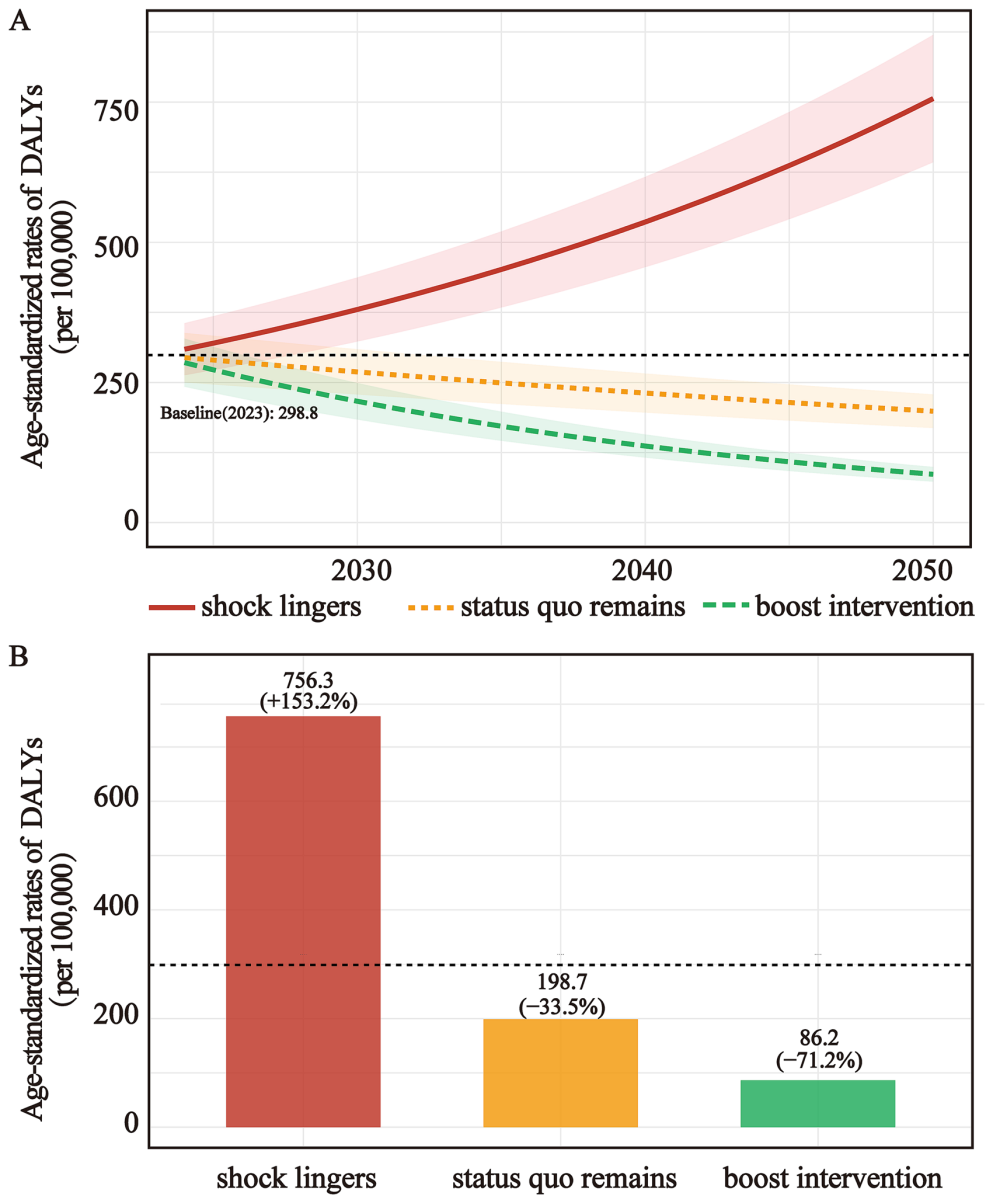
Exploratory scenario estimates of global cervical cancer burden to 2050. **(A)** Temporal trends under three scenarios (shock persistence, status quo, enhanced intervention). **(B)** Comparative outcomes for 2050. These are exploratory scenario models, not robust epidemiological forecasts. Projections assume constant effect sizes, no future disruptions, and linear recovery patterns. DALYs: Disability-adjusted life years.

Without enhanced interventions, pandemic-induced disruptions could substantially delay progress toward the WHO 90–70-90 targets in low-resource settings. Projected global cervical cancer DALY burdens for 2050, based on 2023 data and under three scenarios (shock lingers, status quo remains, and boost intervention), are presented in Figure 5. Under constant EAPC assumptions and a 2023 baseline of 298.8 per 100,000, the shock lingers scenario projected a 153% burden increase to ~750 per 100,000 (95% CI: 640–870). The status-quo scenario projected a decline to ~200 per 100,000 (170–230), while the boost-intervention scenario, aligning with WHO targets, projected a reduction to ~85 per 100,000 (60–110), approaching the 90–70-90 target threshold. Under current trends, low SDI regions will experience a delay of approximately 10–12 years in achieving the WHO 90–70-90 rate targets relative to the 2030 deadline. The ultimate elimination goal (incidence below 4 per 100,000) is likely to be delayed by a decade or more beyond that.

## 4 Discussion

This study evaluated changes in global cervical cancer burden before and during the COVID-19 pandemic, with a focus on the role of health system resilience as reflected by COVID-19 vaccination rate. Our findings provide three main contributions.

### 4.1 The pandemic reversed global progress, with widening inequalities

We found that the COVID-19 pandemic was associated with a reversal of the pre-pandemic declining trend in global cervical cancer incidence indicators. The global ASIR increased by 6.0% during 2020–2023, with EAPC shifting from −0.41% per year to 3.93% per year. This reversal was concentrated in low-SDI regions, where ASIR rose by 18.6% and EAPC increased from −0.21 to 6.87% per year. In contrast, high-SDI regions maintained declining trends. These findings are consistent with previous regional studies that documented short-term screening disruptions, providing global evidence that these disruptions were associated with measurable changes in reported burden indicators (9–11). However, we caution that the pandemic-period EAPCs are based on only 4 years of data. These estimates should be interpreted as descriptive summaries, not stable long-term trends. The observed ASIR increases may partially reflect diagnostic delays and screening backlogs rather than a true biological surge in incidence, given the long latency of cervical cancer.

### 4.2 Distinguishing detection from true burden increases

An important distinction must be made between increases in disease detection and increases in true disease burden. In high-SDI regions, ASIR showed modest changes (EAPC 0.20%, *p* = 0.747) while DALY rates declined substantially (−23.4%). This pattern suggests that the pandemic was not associated with a true increase in cervical cancer burden in these settings; rather, it may reflect temporary screening interruptions followed by catch-up screening that detected prevalent cases. In low-SDI regions, however, ASIR increased sharply (18.6%) while DALY rates remained stable or slightly rose (7.0%, *p* = 0.092). This combination is more concerning and may indicate true burden increases associated with sustained service disruptions. These findings underscore the need for targeted catch-up interventions in low-SDI regions.

### 4.3 Vaccination rate modified the HDI–burden association across development levels

Higher HDI was strongly associated with less burden deterioration (*β* = −15.4, *R*^2^ = 0.259), confirming that development level was associated with pandemic resilience. However, this association was modified by vaccination rate. We identified a significant interaction between HDI and COVID-19 vaccination rates (*β* = 36.1, *p* < 0.001, ΔR^2^ = 6.4%). Stratified analyses revealed a pattern of attenuated HDI effects at higher vaccination levels: in low-HDI countries, higher vaccination rate was associated with significantly less burden deterioration (*β* = −7.15, *p* = 0.005); in high-HDI countries, this association was absent or positive (*β* = 6.53, *p* < 0.001). The positive correlation in high-HDI settings may reflect enhanced diagnostic capacity and catch-up screening after early reopening, rather than a true increase in disease burden. This interpretation is supported by the concurrent decline in DALY rates in these regions.

This pattern suggests that emergency response capacity shows stronger negative associations with burden in settings with lower baseline development. In low-HDI settings, the capacity to deploy vaccines likely co-occurs with broader system capabilities: cold-chain logistics, community health worker networks, and resource prioritization during crises. These same capabilities are needed to sustain cervical cancer screening programs (22, 23). In high-HDI settings, well-established systems already co-occur with sufficient buffers, so additional vaccination capacity shows limited marginal association with burden changes.

### 4.4 Comparison with previous studies

Previous studies focused on documenting service disruptions (9–11) or describing recovery in relation to broad factors such as “political will” or “health investment” (24). Our study offers additional evidence in several ways. First, we extend observations to 2023, showing that recovery trajectories have diverged further since 2021. Second, we provide a measurable proxy indicator—COVID-19 vaccination rate—that is available for 204 countries, far exceeding the rate of conventional health system metrics. Third, we quantify the interaction between dynamic response capacity (vaccination) and structural development (HDI), offering empirical evidence for the concept of health system resilience.

We acknowledge that HPV vaccination—the primary prevention pillar of the WHO strategy—was also disrupted during the pandemic. School-based HPV vaccination campaigns were paused in many countries (25–27). However, these effects are not yet visible in our data due to the long latency of cervical cancer. Our projections incorporate HPV vaccination scale-up in the enhanced intervention scenario, but the full impact of pandemic-related HPV vaccination delays will only become measurable in the coming decades.

### 4.5 Scenario projections and policy implications

Our projections to 2050 are exploratory scenario models, not robust epidemiological forecasts. They illustrate possible futures under different assumptions, not precise point estimates. Under the status quo scenario, low-SDI regions would achieve the WHO 90–70-90 rate targets approximately 12 years later than high-SDI regions. The ultimate elimination goal (incidence <4 per 100,000) would likely be delayed by a decade or more beyond that. Under the enhanced intervention scenario, the global DALY rate could approach the 90–70-90 target threshold (~85 per 100,000). This suggests that accelerated investments in HPV vaccination, screening, and treatment can narrow the gap, but only if resources are prioritized to the most vulnerable settings. The future pandemic scenario we added highlights the fragility of these gains, even a moderate future disruption would likely be associated with further delays in target achievement in low-resource regions.

The fragility of these projections deserves emphasis. Our exploratory scenarios assume no future health system disruptions after 2023. Yet the COVID-19 experience demonstrates that even moderate shocks—school closures, workforce redeployment, supply chain interruptions—can rapidly erode screening and vaccination coverage in low-SDI regions. A future disruption of approximately 50% of the 2020–2021 magnitude, occurring in 2030 with recovery over 3 years, would likely delay the 90–70-90 targets by an additional 3–5 years in the most vulnerable settings. This underscores that achieving cervical cancer elimination requires not only scaling up interventions but also building resilient health systems capable of sustaining core functions during crises.

### 4.6 Limitations

Several limitations should be acknowledged. Firstly, the GBD model’s estimates for low-SDI countries rely on covariate-based inference rather than direct observation. The observed “increase” from 2020 to 2023 may partially reflect algorithmic adjustments rather than true data. Secondly, vaccination rate is an imperfect proxy for health system resilience. It captures response capacity but not preparedness or recovery. It is influenced by vaccine supply, international donations, political governance, and public trust—factors beyond health system capacity. Unmeasured confounders—including baseline HPV vaccination rate, HIV prevalence, and healthcare expenditure—may be associated with both vaccination rates and cervical cancer outcomes. We included HDI to account for structural factors, but residual confounding cannot be excluded. Thirdly, the pandemic-period EAPCs are based on only 4 years of data (2020–2023). High R^2^ values likely reflect mathematical artifacts of fitting a line to a short series rather than strong model fit. These estimates should be interpreted as descriptive summaries of recent changes, not stable long-term trends. Fourthly, we cannot distinguish the relative contributions of screening maintenance versus treatment maintenance, which have different time lags in their effects on ASIR and DALYs. Finally, this study is ecological; associations observed at the national level may not hold at the individual level. Causal inference is precluded.

## 5 Conclusion

The COVID-19 pandemic coincided with increased cervical cancer burden indicators in low-SDI regions. Vaccination rate showed stronger negative associations with burden in low-HDI settings, suggesting emergency mobilization capacity may offset development deficits. Without enhanced interventions, low-SDI regions face substantial delays in achieving WHO 90–70-90 targets.

## Data Availability

The datasets presented in this study can be found in online repositories. The names of the repository/repositories and accession number(s) can be found at: The information utilized in this research is sourced from the 2023 edition of the GBD study, which can be accessed online at http://ghdx.healthdata.org/gbd-results-tool.

## Footnotes

1
http://ghdx.healthdata.org/gbd-results-tool

2
https://hdr.undp.org/data-center

3
https://data.who.int/dashboards/covid19
