# Supplementary material for: COVID-19 vaccination rates as a proxy for health system resilience: implications of development disparities for cervical cancer control during the pandemic
Source: Front Med (Lausanne). 2026 Jul 24;13:1858206. doi: 10.3389/fmed.2026.1858206 (PMC13447365; doi:10.3389/fmed.2026.1858206)
Supplement: Supplementary file 1 [file Table_1.docx]

**Supplementary Materials**

**Table S1. Regression diagnostics for the interaction model**

| **Diagnostic test** | **Statistic** | **Value** | **Interpretation** |
| --- | --- | --- | --- |
| **Multicollinearity** |  |  |  |
| HDI (mean-centered) | GVIF^(1/2Df) | 1.00 | No multicollinearity |
| Vaccination rate (mean-centered) | GVIF^(1/2Df) | 1.00 | No multicollinearity |
| **Residual normality** |  |  |  |
| Shapiro–Wilk test | W | 0.924 | *P* < 0.001; non-normal residuals |
| **Homoscedasticity** |  |  |  |
| Breusch–Pagan test | BP | 5.21 | *P* = 0.157; homoscedasticity assumed |
| **Influential points** |  |  |  |
| Cook's distance | Minimum | 6.79 × 10⁻⁷ | No influential points (all < 0.5) |
|  | Maximum | 0.178 |  |
|  | > 0.5 | 0 |  |

**Note:** Due to non-normality of residuals, Huber-White robust standard errors were reported for all regression coefficients. GVIF, generalized variance inflation factor; HDI, Human Development Index.

**Figure S1. Scatter plot of HDI and COVID-19 vaccination rate.**

**Figure S2. Regression diagnostic plots for the interaction model (EAPC of DALYs ~ HDI × COVID-19 vaccination rate, n = 173).** (A) Residuals vs Fitted: Random scatter supports linearity. (B) Normal Q-Q Plot: Deviations indicate non-normality (Shapiro–Wilk *P* < 0.001); robust standard errors applied. (C) Scale-Location Plot: No trend supports homoscedasticity (Breusch–Pagan *P* = 0.157). (D) Residuals vs Leverage: No influential points (max Cook's distance = 0.178).
